# Assessing the Quality of Mobile Exercise Apps Based on the American College of Sports Medicine Guidelines: A Reliable and Valid Scoring Instrument

**DOI:** 10.2196/jmir.6976

**Published:** 2017-03-07

**Authors:** Yi Guo, Jiang Bian, Trevor Leavitt, Heather K Vincent, Lindsey Vander Zalm, Tyler L Teurlings, Megan D Smith, François Modave

**Affiliations:** ^1^ Institute of Natural Resources and Environmental Audits Nanjing Audit University Nanjing China; ^2^ Department of Health Outcomes and Policy University of Florida Gainesville, FL United States; ^3^ Department of Orthopaedics and Rehabilitation University of Florida Gainesville, FL United States

**Keywords:** mHealth, mobile apps, physical activity, measures

## Abstract

**Background:**

Regular physical activity can not only help with weight management, but also lower cardiovascular risks, cancer rates, and chronic disease burden. Yet, only approximately 20% of Americans currently meet the physical activity guidelines recommended by the US Department of Health and Human Services. With the rapid development of mobile technologies, mobile apps have the potential to improve participation rates in exercise programs, particularly if they are evidence-based and are of sufficient content quality.

**Objective:**

The goal of this study was to develop and test an instrument, which was designed to score the content quality of exercise program apps with respect to the exercise guidelines set forth by the American College of Sports Medicine (ACSM).

**Methods:**

We conducted two focus groups (N=14) to elicit input for developing a preliminary 27-item scoring instruments based on the ACSM exercise prescription guidelines. Three reviewers who were no sports medicine experts independently scored 28 exercise program apps using the instrument. Inter- and intra-rater reliability was assessed among the 3 reviewers. An expert reviewer, a Fellow of the ACSM, also scored the 28 apps to create criterion scores. Criterion validity was assessed by comparing nonexpert reviewers’ scores to the criterion scores.

**Results:**

Overall, inter- and intra-rater reliability was high with most coefficients being greater than .7. Inter-rater reliability coefficients ranged from .59 to .99, and intra-rater reliability coefficients ranged from .47 to 1.00. All reliability coefficients were statistically significant. Criterion validity was found to be excellent, with the weighted kappa statistics ranging from .67 to .99, indicating a substantial agreement between the scores of expert and nonexpert reviewers. Finally, all apps scored poorly against the ACSM exercise prescription guidelines. None of the apps received a score greater than 35, out of a possible maximal score of 70.

**Conclusions:**

We have developed and presented valid and reliable scoring instruments for exercise program apps. Our instrument may be useful for consumers and health care providers who are looking for apps that provide safe, progressive general exercise programs for health and fitness.

## Introduction

### Physical Activity

Regular physical activity provides many health benefits [1-3] and is one of the recommendations made to address epidemic lifestyle-related diseases in the United States [4,5]. Participation in regular physical activity can lower the risk of early death and many diseases, including coronary heart disease, stroke, adverse blood lipid profile, type 2 diabetes, metabolic syndrome, some cancers, obesity, hypertension, bone and joint diseases, some autoimmune conditions, and depression [1,6-9]. Prospective epidemiological studies have documented a causal relationship between physical inactivity and heart disease, the leading cause of death in the United States [10-16]. These studies show that individuals who are more physically active have lower rates of heart disease, and the most physically active group develops heart disease at rates half of that of the most sedentary group [17,18]. However, despite the many benefits of regular physical activity, the majority of US adults do not meet the national physical activity guidelines [6,19,20]. It is estimated that only 20.9% of US adults meet the recommendations for both aerobic and muscle-strengthening activities [6].

In the past decade, there has been a tremendous increase in the availability and use of mobile phones and mobile phone apps [21,22]. A 2015 survey shows that 64% of US adults and 82% of US adults aged between 18 and 49 years own an app-enabled mobile phone [21]. This increase in mobile phones use has allowed for a growth in mobile phone apps related to physical activity and exercise. There is an estimated 100,000 health- and fitness-related apps in the Apple store alone, and over 165,000 apps when including the Android’s Google Play store [23]. These apps include fitness and exercise trackers such as heart rate monitors, step counters, exercise programs, and coaching apps. There is preliminary evidence that these apps can be used effectively to improve health-related behaviors for a variety of chronic conditions [22,24-40]. However, there are very few exercise program apps that are evidence-based and follow the exercise guidelines set forth by the American College of Sports Medicine (ACSM) [41,42].

### Three Components of Exercise Programs

The US Department of Health and Human Services recommends that adults should perform at least 150 min of moderate-intensity aerobic activity or 75 minutes of vigorous-intensity activity per week in addition to performing muscle-strengthening activities at least two times per week [43]. Further, based on the cumulative evidence pertaining to health and fitness, the ACSM recommends that the frequency, intensity, time, and type (FITT) principle should be followed for any exercise program to have health benefits while avoiding injuries and other adverse events. Exercise sessions should include components of safety precautions, warm-up, conditioning including strengthening, and cool-down. In addition, exercise programs should progress safely at a rate that is appropriate for the individual’s fitness level and goals. The ACSM principles of exercise prescription recommend that optimal exercise programs include 3 main components: aerobic exercise, strength and resistance exercise, and flexibility. These components improve cardiovascular fitness, strength, neuromuscular fitness, and overall health [42].

Presently, the quality and accuracy of available mobile exercise program apps and the theoretical foundations that underpin these apps are not clear. Modave et al [41] used an initial scoring system to determine whether the content of free mobile apps related to exercise programming were evidence-based and adhered to the ACSM guidelines for aerobic exercise, strength and resistance exercise, and flexibility. These findings revealed a significant gap in the app content with the ACSM guidelines. A standardized instrument developed from the system in the first study that determines the quality of exercise programming apps could be of widespread benefit to clinicians and consumers who must make informed decisions about which apps to choose. We address this significant problem by developing a reliable and valid scoring instrument that can evaluate the quality of fitness and exercise-prescriptive apps with respect to the highest standards set forth by the ACSM. To our knowledge, there are no fitness app scoring instruments developed based on the ACSM exercise guidelines. Stoyanov et al developed a rating scale for assessing the quality of mobile health apps in general [44]. But this scale assesses domains of engagement, functionality, aesthetics, and information quality, rather than evidence-based exercise principles. In this paper, we describe our process of instrument development and present data demonstrating the inter- and intra-rater reliability and criterion validity of the instrument. Finally, the most popular free exercise prescriptive apps are scored and ranked using the developed instrument.

## Methods

### Focus Groups and Instrument Development

Our study included two focus groups (N=14) from whom we elicited input and guidance on survey item refinement and questionnaire design. Specifically, we asked the focus group participants to identify unclear words or sentences, suggest alternative ways of phrasing a question, recommend response formats, and consider how the questions would have worked in eliciting responses. University of Florida (UF) College of Medicine employees and students were recruited to participate in these focus groups.

Before the focus groups, the study investigators wrote survey questions according to the published ACSM exercise principles to create an initial version of the scoring instrument. (ACSM Guidelines, 9th ed.) For example, the single training session principle for aerobic exercise “Warm-up: 5-10 minutes of light/moderate intensity cardiovascular exercise” was written as “Does the app advise you to warm up for 5-10 minutes with light or moderate cardiovascular exercise before starting any aerobic exercise?” The responses were initially written on a 3-point scale with 0 indicating “Principle missing,” 1 indicating “Principle present but unclear (or not 100% aligned with ACSM guidelines),” and 2 indicating “Principle present and clear.” The scoring instrument was divided into 3 sections: (I) aerobic exercise, (II) strength and resistance, and (III) flexibility. Sections I and II were further divided into 3 subsections: (1) safety, (2) program principles, and (3) single training session principles. Section III was divided into 2 subsections: (1) safety and (2) program principles.

Next, we provided the participants with the initial instrument and asked them to independently rate 5 fitness apps randomly selected from the apps evaluated in [41]. We instructed the participants to list the problems of the instrument during app scoring. All participants were allowed at least one day to thoroughly examine the instrument. During the focus groups, our moderator went through each question in the instrument and asked the participants to discuss potential issues such as ambiguity, excessive complexity, or inaccuracy with the text, phrasing, and format of the questions and accompanying responses. For each item, we asked the participants if they could paraphrase the question or if they thought the question should be asked in another way. Following completion of the focus groups, the investigators met to review the findings and to develop the final scoring instrument. The final instrument consisted of 27 questions, with 10 questions on aerobic exercise, 12 questions on strength and resistance, and 5 questions on flexibility (see Multimedia Appendix 1).

### Scoring Strategy

The 3 main components of the ACSM exercise principles (aerobic exercise, strength and resistance, and flexibility) were weighted 3:3:1 based on the time allocated by the ACSM within a standard exercise program for health and fitness. For each of the 3 principles, the subsections (safety, program principles, and single training session principles) were allocated the same weight due to the lack of evidence that the subsections should be emphasized differently. Therefore, the overall quality score was scaled to have a highest possible score of 70, with 30 points in aerobic exercise, 30 points in strength and resistance, and 10 points in flexibility.

### Data Collection and Psychometric Analysis

#### Sample

For testing the psychometric properties of the scoring instrument, we chose the same fitness apps evaluated in [41]. The apps were selected by searching the Apple store with keywords “workout” and “training” in the “health and fitness” category, and selecting the top 50 apps, based on their popularity. After removing duplicate apps from these two search terms, a list of 83 apps was generated. The investigators then evaluated and removed apps that did not provide exercise prescriptive programs. Finally, 30 apps were selected for scoring in [41]. During the development phase of the new instrument, two of the apps were no longer available in the app store. Therefore, we used the remaining 28 apps for scoring and psychometric analysis.

#### Inter- and Intra-Rater Reliability

We assessed the inter-rater and intra-rater (test-retest) reliability of the instrument. To assess inter-rater reliability, we asked 3 reviewers to score all 28 apps using the instrument concurrently but independently. The reviewers included 1 staff member and 2 college students from the UF Orthopaedics and Sports Medicine Institute. To assess intra-rater reliability, 5 apps were randomly selected from the 28 apps (7 Minute Workout, Body Space, FitStar, JEFIT, and Sworkit). Then, the same 3 reviewers rated the 5 apps again using the instrument approximately 1 month later. The Spearman correlation coefficient *r* among pairs of scores was computed as a measure of reliability.

#### Criterion Validity

We asked the director of UF Human Performance Laboratory and UF Health Sports Performance Center (expert reviewer, Fellow of the ACSM) to rate all 28 apps on a scale of 1-10 (criterion scores). In addition, the expert reviewer rated 5 apps (7 Minute Workout, Body Space, FitStar, JEFIT, and Sworkit) using our scoring instrument. Criterion validity was then assessed by calculating the Spearman correlation coefficient between the criterion scores and those obtained with the instrument, given by the nonexpert reviewers. For the 5 apps rated using the instrument, we computed the Spearman correlation coefficient between scores from the expert and nonexpert reviewers. In addition, we computed a weighted kappa statistic to assess the agreement between the expert and nonexpert reviewers for each app. We used the following guidelines for interpreting kappa statistics suggested by Landis and Koch: <0=poor agreement, 0-.2=slight agreement, .2-.4=fair agreement, .4-.6=moderate agreement, .6-.8=substantial agreement, and .8-1=almost perfect agreement [45].

## Results

### Instrument Item Development

The focus groups identified multiple challenges when using the initial instrument for scoring. The biggest challenge was the need to clarify meanings and definitions of domain-specific words and phrases. For instance, one of the questions asked was “Does the app advise you to train each major muscle group 2-3 times per week?” Focus group participants did not know what the “major muscle groups” were or what “advise” represented and thus could not judge whether the app provided a comprehensive strength training program or not. In addition, some participants did not understand some of the technical terms such as metabolic equivalent (MET) and Proprioceptive Neuromuscular Facilitation (PNF) stretching. To improve comprehension, participants were asked to suggest alternative strategies for phrasing the questions. Eventually, we removed terms that were too technical and added footnotes to most questions explaining the words that were unclear and phrases compiled from the focus groups.

Participants’ preferences of response options were tested. The majority of the participants felt that the initial 3-point response scale did not fully differentiate the apps on quality since there was only one middle category “Principle present but unclear (or not 100% aligned with ACSM guidelines).” As a result, final response options were revised to include a 5-point Likert scale with 1 being “No,” 3 being “Partially,” and 5 being “Yes.”

### Inter- and Intra-Rater Reliability

Levels of inter-rater reliability for the principle section, subsection, and overall scores for each pair of reviewers are summarized in Table 1. Reliability was high overall, with most coefficients being greater than .7. The average inter-rater reliability was .88 for R1-R2, .81 for R1-R3, and .73 for R2-R3. The reliability coefficients range from .59 to .99, and all coefficients were statistically significant. The least reliable question pertained to safety warning for aerobic exercise and asked, “Does the app provide safety warnings about health conditions or advise you to consult a doctor before starting any aerobic exercise?” (Section I.1.). The average inter-rater reliability across the rater pairs was .69 for this safety warning question. Overall, the aerobic exercise (average *r*=.75) and strength and resistance (average *r*=.79) sections had lower reliability than the flexibility section (average *r*=.88).

**Table 1:** Inter-rater reliability for section, subsection, and overall scores for each rater pair.

Section		R1-R2	*P* value	R1-R3	*P* value	R2-R3	*P* value	Average
**I. Aerobic exercise**		.80	<.001	.76	.009	.70	.011	.75
	1. Safety	.77	.002	.68	.01	.62	.012	.69
	2. Program principles	.76	.003	.89	<.001	.82	.001	.82
	3. Single training session principles	.92	<.001	.92	<.001	.81	.001	.88
**II. Strength and resistance**		.94	<.001	.73	<.001	.69	<.001	.79
	1. Safety	.92	<.001	.77	<.001	.65	<.001	.78
	2. Program principles	.95	<.001	.92	<.001	.84	<.001	.90
	3. Single training session Principles	.84	<.001	.78	<.001	.59	.003	.74
**III. Flexibility**		.97	<.001	.87	.009	.81	.009	.88
	1. Safety	.99	<.001	.90	<.001	.88	.002	.92
	2. Program principles	.87	.005	.70	.02	.67	.03	.75
Overall score		.92	<.001	.85	<.001	.79	<.001	.85
Average		.88		.81		.73		

Results from the intra-rater (test-retest) reliability analysis are summarized in Table 2. Overall, intra-rater reliability was high with most coefficients being greater than .7. The reliability coefficients ranged from .47 to 1.00, and all reliability coefficients were statistically significant. JEFIT had the lowest intra-rater reliability among the apps (average *r*=.66). The reliability for Reviewer 2 was only 0.47 for JEFIT. On the other hand, the intra-rater reliability was high for FitStar. All raters were able to provide the same scores for FitStar 1 month later.

**Table 2:** Intra-rater (test-retest) reliability by rater.

App	R1	*P* value	R2	*P* value	R3	*P* value	Average
7 Minute Workout	1.00	-	.85	<.001	.74	.006	.86
Body Space	.64	<.001	.76	<.001	.85	<.001	.75
FitStar	1.00	-	1.00	-	1.00	-	1.00
JEFIT	.75	<.001	.47	.01	.76	<.001	.66
Sworkit	.69	<.001	.73	<.001	.76	<.001	.73

### Criterion Validity

In evaluating the validity of the instrument, the Spearman correlation coefficient between the criterion scores and those obtained with the instrument was .70 (*P*<.001) for Reviewer 1, .69 (*P*<.001) for Reviewer 2, and .78 (*P*<.001) for Reviewer 3. The Spearman correlation coefficient between the criterion scores and average nonexpert reviewer scores obtained with the instrument was .72 (*P*<.001). The high correlations provided support for the validity of our scoring instrument.

Table 3 summarizes weighted kappa statistic *κ* and Spearman correlation coefficient *r* from our agreement analysis. The weighted kappa statistics ranged from .67 to .99, indicating a substantial (*κ* is between .6 and .8) or almost perfect (*κ* is between .8 and 1) agreement between the scores given by the expert and nonexpert reviewers. The correlations between the scores from the nonexpert reviewers and those from the expert reviewer were high, ranging from .58 to .99, with most coefficients being greater than .7. The agreement was relatively poorer for FitStar (average *κ*=.73; average *r*=.76) and Sworkit (average *κ*=.79; average *r*=.76) when compared with the other apps.

**Table 3:** Validity measures using weighted kappa statistic *κ* and Spearman correlation coefficient *r*.

App	R1-Expert		R2-Expert		R3-Expert		Average	
	*κ* (*P* value)	*r* (*P* value)	*κ* (*P* value)	*r* (*P* value)	*κ* (*P* value)	*r* (*P* value)	*κ*	*r*
7 Minute Workout	.94 (<.001)	.74 (.005)	.84 (.002)	.58 (.04)	.94 (<.001)	.74 (.008)	.91	.69
Body Space .81 (<.001)	.81 (<.001)	.67 (.005)	.84 (<.001)	.78 (<.001)	.77 (<.001)	.75	.81	
FitStar	.68 (.003)	.75 (<.001)	.83 (<.001)	.79 (<.001)	.68 (.001)	.75 (<.001)	.73	.76
JEFIT	.98 (<.001)	.99 (<.001)	.91 (<.001)	.94 (<.001)	.88 (<.001)	.88 (<.001)	.92	.94
Sworkit	.78 (.001)	.69 (<.001)	.86 (<.001)	.82 (<.001)	.72 (.001)	.76 (<.001)	.79	.76

### Overall and Section Quality Sores

The overall and section quality scores for the 28 apps evaluated using the scoring instrument are summarized in Table 4. The apps are presented in order from high to low overall scores. None of the apps had an overall quality score higher than 35 out of a highest possible score of 70 points. This confirms the results reported in [41] that the most popular apps for exercise prescription do not meet the standards set forth by the ACSM. Among the 28 apps, only 4 apps had an overall score higher than 30 (The Johnson and Johnson Official 7-Minute Workout, 33.5; Nike+ Training Club, 32.6; Running for Weight Loss: Interval Training, 32.6; Fitness Buddy Free, 31.0). For the aerobic exercise principle section, Running for Weight Loss: Interval Training (section score=26.7) was the only app that had a quality score higher than 20, out of a highest possible score of 30 points. For the strength and resistance principle section, 3 apps had a quality score higher than 20, out of a highest possible score of 30 points (StrongLifts 5x5, 24.0; Fitness Buddy Free, 22.9; FitnessBuilder, 21.2). For the flexibility principle section, 3 apps had a quality score higher than 5, out of a highest possible score of 10 points (FitnessBuilder, 7.1; Simply Yoga Free, 6.4; Daily Yoga-Lose Weight, Get Relief, 6.2). The average overall quality score was 17.8 (SD 8.9). The average section quality scores were 11.7 (SD 5.9) for aerobic exercise, 13.9 (SD 5.2) for strength and resistance, and 4.0 (SD 1.9) for flexibility.

**Table 4:** Final scores of the apps evaluated with the scoring instrument. All values are expressed in points.

Rank	App	Overall^a^	Section I^b^	Section II^c^	Section III^d^
1	The Johnson and Johnson Official 7-Minute Workout	33.5	16.3	17.2	–
2	Nike+ Training Club	32.6	10.0	19.5	3.1
3	Running for Weight Loss: Interval Training	32.6	26.7	–	3.5
4	Fitness Buddy Free	31.0	8.1	22.9	–
5	FitnessBuilder	28.3	–	21.2	7.1
6	JEFIT	27.5	8.4	16.3	2.8
7	Body Space	26.2	9.0	14.9	2.4
8	Daily Workouts Free	25.5	13.0	12.5	–
9	StrongLifts 5x5	24.0	–	24.0	–
10	Jillian Michaels Slim Down	19.9	–	19.9	–
11	Fitness Point-Workout Exercise	19.0	–	19.0	–
12	C25K-5K Trainer Free	17.7	17.7	.	–
13	7 Minute Workout	17.5	8.7	8.8	–
14	Sworkit	17.5	7.0	8.0	2.5
15	7-Minute Workout-Fitness for Women	16.9	7.0	9.9	–
16	Abs Workout: Get Your Six Pack	14.9	–	14.9	–
17	Daily Butt Workout Free	14.9	–	14.9	–
18	Instant Abs Trainer	12.2	–	12.2	–
19	FitStar	11.3	–	8.9	2.4
20	Daily Ab Workout Free	11.3	–	11.3	–
21	Workout Trainer	10.0	–	10.0	–
22	Runtastic Six Pack Abs Trainer	9.1	–	9.1	–
23	The 7 Minute Workout-Get Fit	8.4	–	8.4	–
24	Cardio-Heart Rate Monitor+7 Minute Workout	8.4	–	8.4	–
25	Belly Fat Workout Free	8.3	–	8.3	–
26	Strava Running and Cycling	8.0	8.0	–	–
27	Simply Yoga Free	6.4	–	–	6.4
28	Daily Yoga-Lose Weight, Get Relief	6.2	–	–	6.2
	Mean (SD)	17.8 (8.9)	11.7 (5.9)	13.9 (5.2)	4.0 (1.9)

^a^The highest possible overall score is 70; the highest possible sections I and II score is 30; the highest possible section III score is 10.

^b^Section I is aerobic exercise.

^c^Section II is strength and resistance.

^d^Section III is flexibility.

All subsection scores for the 28 apps evaluated in this study are summarized in Multimedia Appendix 2. Although the scores were low in general, the apps performed better when providing safety precautions and describing program principles for strength and resistance training than for aerobic exercise. Among apps with a strength and resistance component, more than half (12 out of 23, 52%) of them scored at least 5 points out of a maximal of 10 points in the safety subsection, and 9 of them (39%) scored at least 5 points out of a maximal of 10 points in the program principles subsection. On the other hand, among apps with an aerobic exercise component, 3 out of 12 apps (25%) scored higher than 5 points in the safety subsection, and only 2 apps (17%) scored higher than 5 points in the program principles subsection.

## Discussion

### Principal Findings

We developed and presented a novel scoring instrument for evaluating the quality of exercise program apps with respect to the ACSM exercise prescription guidelines. Inter- and intra-rater reliability and criterion validity were assessed using the Spearman correlation coefficients and weighted kappa statistics. Our results showed excellent reliability and validity of our instrument, indicating that it provides accurate and stable measurement of the quality of exercise apps. In addition, our findings confirmed the preliminary study of Modave et al that very few of the most popular exercise prescriptive apps are of sufficient quality to provide evidence-based exercise prescription. This is a significant problem, particularly for novice exercisers who do not have the necessary expertise to develop their own program or assess whether a program is well-designed. In addition, the subscores pertaining to the aerobic, strength and resistance, and flexibility components suggest that most apps are of substandard quality even if we assume that they were designed to address a single aspect of exercise prescription.

For inter-rater reliability, the least reliable question asked whether an app provides any safety warning before starting aerobic exercise. One potential reason for the low reliability is that fitness apps have a variety of ways of delivering safety warnings. Current fitness apps are very sophisticated in terms of user interface design and content presentation. Safety warnings may be presented as part of the introductory text that users need to read or delivered as a push notification that users must read and close before starting a training program. Furthermore, safety warnings may be presented as part of the exercise instructional videos, as many apps are delivering training programs in videos. This diversity in safety warning delivery methods creates some difficulty for app reviewers to find and judge the appropriateness of safety warnings, especially in sophisticated fitness apps.

Although the overall intra-rater reliability is high, we have observed a few relatively lower reliability coefficients. Given that the test and retest were conducted 1 month apart, it is possible that some of the app contents and functions were updated, introducing additional unexpected measurement errors from the app reviewers. In order to survive and stay competitive in the crowded health and fitness apps market, app companies are constantly redesigning their product. It is not surprisingly to see fitness apps redesigning user interface, adding or removing functions, or disappearing from the app stores.

The overall quality scores and principle section quality scores are low for the apps evaluated in this study. It indicates that most popular fitness apps do not fully follow the evidence-based exercise principles set forth by the ACSM. On the other hand, one reason for the low overall quality scores is that not all apps have all 3 components of aerobic exercise, strength and resistance exercise, and flexibility. As the number of fitness apps continue to grow, they have become more specialized and focused in functions, with many apps providing exercise programs only for one specific type of exercise. For instance, with a score of 24.0 (out of 30.0 points) for the strength and resistance section, StrongLifts 5x5 is an app of good quality for strength training. However, it does not provide sufficient prescriptive principles for the aerobic exercise and flexibility, making the overall quality score relatively low for this app. Therefore, we recommend that the section quality scores always be evaluated with the overall quality score, which provides a more comprehensive evaluation of the app quality. In addition, app designers may need to consider all 3 main components of the ACSM exercise prescription principles when designing exercise apps, which can improve the degree to which apps adhere to the ACSM standards.

### Limitations

One limitation of the study is that evaluating the quality of fitness apps using the instrument in its current form can be time consuming. Our reviewers have reported spending on average 30-40 min to score one app. It can be tedious when a large number of apps need to be evaluated. However, the reviewers have also noted that the scoring process becomes fairly automatic after having reviewed a few apps. Thus, the scoring process can be shortened with some practice. However, future research is needed to develop reliable and valid short forms of the instrument. Another limitation of the study is the small sample size for intra-rater reliability testing. More apps need to be reviewed for computing and analyzing section-specific intra-rater reliability. Nonetheless, reliability coefficients from this study are all statistically significant, providing initial evidence that the instrument is reliable. Finally, we acknowledge that this scoring instrument was designed to capture app quality for overall exercise programs, not for specialized programs that emphasize one type of activity such as weight lifting, yoga, or running. Additional scoring methods are needed to determine quality for these specialized exercise programs.

### Conclusions

We have developed a reliable and valid instrument for evaluating the quality of exercise program apps according to the ACSM exercise prescription guidelines. This instrument can be used to determine which apps are useful for novice exercisers and health care providers who are looking for apps that provide safe, progressive general exercise programs for health and fitness. Given that the instrument provides separate scores for the 3 main components of ACSM principles, it can also be used to determine which apps are suitable for each of the components of an exercise routine.

In future research, we plan to examine the relationship between scores from our instrument and those from other instruments for evaluating app quality, including the instrument published by Stoyanov et al. This would provide a more comprehensive evaluation of exercise apps and thus help users to select apps based on not only evidence-based exercise principles but also functionality and aesthetics. In addition, we plan to evaluate the relationship between app quality scores and behavioral outcomes (eg, adherence, exercise, and health outcomes) among app users. It is important to quantify the differential impacts of app quality aspects on these outcomes to design better exercise apps.

## Appendix

Multimedia Appendix 1

Multimedia Appendix 2

## Abbreviations

ACSM: American College of Sports Medicine
FITT: frequency, intensity, time, and type
MET: metabolic equivalent
PNF: proprioceptive neuromuscular facilitation
SD: standard deviation
UF: University of Florida
