# Supplementary material for: Assessing the Quality of Mobile Exercise Apps Based on the American College of Sports Medicine Guidelines: A Reliable and Valid Scoring Instrument
Source: J Med Internet Res. 2017 Mar 7;19(3):e67. doi: 10.2196/jmir.6976 (PMC5360889; doi:10.2196/jmir.6976)
Supplement: Multimedia Appendix 1 [file jmir_v19i3e67_app1.pdf]

FITNESS APP SCORING INSTRUMENT

The American College of Sports Medicine (ACSM) recommended exercise prescription consists of three main components: aerobic exercise, strength and resistance, and flexibility. Each component contains safety, programming (the FITT principle: frequency, intensity, time, and type), and single-session principles.

IMPORTANT:

1. **Please install the app on your mobile phone and thoroughly examine all app components.**
2. **If the app has multiple exercise levels, please rate the "beginner" or "starting" level.**
3. **If the app has multiple exercise routines with text or video instructions, please randomly examine four routines and provide an overall judgement.**
4. **Give a partial score if the app does not explicitly in words or audio advise you to follow the ACSM exercise principles (FITT principles; session components of warmup, conditioning, cooldown; progression; specific safety instructions or warnings), but provided some presentation of principles through demonstration or program set-up.**

Part I. Aerobic Exercise (Cardio)

Does the app have an aerobic exercise component?

☐ **Yes** ☐ **No** (Skip to Part II if the answer is No)

A. Safety

1. Does the app provide safety warnings about health conditions or advise you to consult a doctor before starting any aerobic exercise?

No		Partially		Yes
1	2	3	4	5

Note: Safety warnings in disclaimers do not count – some apps may have safety warnings at the beginning of workout videos.

B. Program Principles

1. Does the app advise you to perform aerobic exercise 3–5 times per week?

No		Partially		Yes
1	2	3	4	5

Note: Give a partial score if the app does not explicitly instruct you to perform aerobic exercise 3–5 times per week but allows choosing exercise frequency in the program.

2. Does the app advise you to monitor the intensity of aerobic exercises by monitoring heart rate?

No		Partially		Yes
1	2	3	4	5

Note:

1. Other methods for measuring aerobic exercise intensity include the perceived exertion methods in which you may be asked "How hard are you working on a scale of 1-10 or 6-20 points?"
2. Give a partial score if the app does not explicitly instruct you but allows you to monitor the intensity.

3. Is the workout duration appropriate for the aerobic exercises?				
No		Partially		Yes
1	2	3	4	5

Note: ACSM recommends adults get ≥150 minutes of moderate (or ≥75 minutes of vigorous) intensity exercise per week. This can be met through 30–60 minutes of moderate (or 20–60 minutes of vigorous) intensity exercise per day.

4. Does the app advise you to engage in different types of aerobic exercises?				
No		Partially		Yes
1	2	3	4	5

Note:
1. Give a partial score if the app does not explicitly instruct you to engage in different types of aerobic exercises but provides/demonstrates different types of aerobic exercises in the workout routines.
2. Examples of aerobic exercises include but are not limited to walking, running, swimming, cycling, and rowing.

5. Does the app advise you to progress appropriately for aerobic exercises?				
No		Partially		Yes
1	2	3	4	5

Note:
1. Give a partial score if the app does not explicitly instruct you how to progress appropriately, but there are signs of appropriate progression in the workout routines.
2. ACSM recommends increasing time 5–10 minutes per session every 1–2 weeks until proper duration values are met. Do not exceed 5–10% increase in total time per week.

C. Single Training Session Principles

1. Does the app advise you to warm up before starting any aerobic exercise?				
No		Partially		Yes
1	2	3	4	5

Note:
1. Give a partial score if the app does not explicitly instruct you to warm up or how to warm up but has warm up activities in the workout routines.
2. ACSM recommends warming up for 5–10 minutes with light or moderate cardiovascular exercise.

2. Does the app advise you on conditioning time during each workout?				
No		Partially		Yes
1	2	3	4	5

Note: ACSM recommends 20–60 minutes of aerobic, resistance, neuromotor, or sport activities; or accumulative bouts of 10 minutes of activity during the day.

**3. Does the app advise you to cool down after aerobic exercise?**				
No		Partially		Yes
1	2	3	4	5

Note:
1. Give a partial score if the app does not explicitly instruct you to cool down or how to cool down but has cool down in the workout routines.
2. ACSM recommends cooling down for 5–10 minutes with light or moderate cardiovascular exercise.

**4. Does the app advise you to stretch after aerobic exercise?**				
No		Partially		Yes
1	2	3	4	5

Note:
1. Give a partial score if the app does not explicitly instruct you to stretch after aerobic exercise but includes stretching activities after aerobic exercise in the workout routines.
2. ACSM recommends stretching for ≥10 minutes after exercise.

Part II. Strength and Resistance

Does the app have a strength/resistance training component?

☐ **Yes** ☐ **No** (Skip to Part III if the answer is No)

A. Safety

**1. Does the app provide safety warnings about strength training?**				
No		Partially		Yes
1	2	3	4	5

Note: Example safety warnings: do not keep exercising if pain develops; perform with proper technique (full range of motion in joint when possible); perform with controlled inhaling during the eccentric phase and exhaling during the concentric phase.

B. Program Principles

**1. Does the app advise you to train each major muscle group 2–3 times per week?**				
No		Partially		Yes
1	2	3	4	5

Note:
1. Give a partial score if the app does not explicitly instruct you to train 2–3 times per week but allows choosing exercise frequency in the program.
2. Give a partial score if the app <u>does not</u> cover all the major muscle groups: quadriceps, hamstrings, calves, chest, back, shoulders, triceps, biceps, forearms, trapezius, and abs.

**2. Does the app clearly state the appropriate intensity of strength training?**				
No		Partially		Yes
1	2	3	4	5

Note: ACSM recommends 60–70% of 1 repetition maximum (1RM) for novice to intermediate trainees; ≥80% of 1RM for experienced trainees; and 40–50% of 1RM for older or sedentary people beginning strength training.

**3. Does the app clearly recommend or support different types of multi-joint exercises in strength training?**				
No		Partially		Yes
1	2	3	4	5

Note: Single-joint exercises can be included but are not required. Using a variety of equipment is not required, but each routine should include all major joints such as the knee, hip, shoulder.

**4. Does the app clearly state or support the appropriate repetitions for strength training?**				
No		Partially		Yes
1	2	3	4	5

Note: ACSM recommends 8–12 repetitions to improve strength for most adults and 10–15 repetitions to improve strength in middle aged and older persons starting exercise.

**5. Does the app clearly state or support the appropriate sets for strength training?**				
No		Partially		Yes
1	2	3	4	5

Note: ACSM recommends 2–3 sets for most adults and 1 set for older and novice trainees.

**6. Does the app clearly advise you to rest for 1–3 minutes between sets and 48 hours between training days?**				
No		Partially		Yes
1	2	3	4	5

Note:
1. Give a partial score if either the between-sets or the between-days rest recommendation is missing.
2. Give a partial score if the app does not explicitly instruct you to rest but has appropriate rests in workout routines.

**7. Does the app clearly advise you to progress gradually each week for greater resistance, repetitions, or frequency?**				
No		Partially		Yes
1	2	3	4	5

Note: Give a partial score if the app does not explicitly instruct you to progress appropriately, but there are signs of appropriate progression in the workout programs.

C. Single Training Session Principles

1. Does the app clearly advise you to warm up before starting any strength training?

No		Partially		Yes
1	2	3	4	5

Note:
1. ACSM recommends warming up for 5–10 minutes with light or moderate aerobic exercise.
2. Give a partial score if the app does not explicitly instruct you to warm up but has warm up in workout routines.

2. Does the app advise you on the "conditioning" of resistance or strength training?

No		Partially		Yes
1	2	3	4	5

Note: ACSM recommends 8-12 exercises of all major muscle groups.

3. Does the app clearly advise you to cool down after strength training?

No		Partially		Yes
1	2	3	4	5

Note:
1. ACSM recommends cooling down for 5–10 minutes with light or moderate aerobic exercise.
2. Give a partial score if the app does not explicitly instruct you to cool down but demonstrates or provides cool down activities in workout routines.

4. Does the app advise you to stretch after strength training?

No		Partially		Yes
1	2	3	4	5

Note:
1. Give a partial score if the app does not explicitly instruct you to stretch after strength training but has stretching activities after strength training in the workout routines.
2. ACSM recommends stretching for ≥10 minutes after exercise.

Part III. Flexibility

Does the app have a flexibility training component?

☐ Yes ☐ No

A. Safety

1. Does the app provide safety warnings about flexibility exercise?

No		Partially		Yes
1	2	3	4	5

Note: Example safety warnings: no bouncing while holding a stretch; use a light warm up with aerobic activity; or use most heat packs to enhance stretching benefit.

B. Program Principles

1. Does the app clearly advise you to perform flexibility exercise a minimum of 2–3 times per week?

No		Partially		Yes
1	2	3	4	5

Note: Give a partial score if the app does not explicitly instruct you to perform flexibility exercise 2–3 times per week but allows choosing stretching frequency in the program.

2. Does the app clearly state the appropriate intensity for flexibility exercises?

No		Partially		Yes
1	2	3	4	5

Note: ACSM recommends stretching to point of tightness with no pain.

3. Does the app clearly state the appropriate volume for flexibility exercises?

No		Partially		Yes
1	2	3	4	5

Note: ACSM recommends 60 seconds per muscle-tendon region of all major joints in the body.

4. Does the app clearly state or support the appropriate pattern for flexibility exercises?

No		Partially		Yes
1	2	3	4	5

Note: ACSM recommends repeating each stretch 2–4 times in each workout session.
