# Supplementary material for: Assessing the Quality of Mobile Exercise Apps Based on the American College of Sports Medicine Guidelines: A Reliable and Valid Scoring Instrument
Source: J Med Internet Res. 2017 Mar 7;19(3):e67. doi: 10.2196/jmir.6976 (PMC5360889; doi:10.2196/jmir.6976)
Supplement: Multimedia Appendix 2 [file jmir_v19i3e67_app2.pdf]

Appendix 2. Final scores of the apps evaluated with the scoring instrument, by section.

Rank	App	Overall	Section I	Section I.A.	Section I.B.	Section I.C.	Section II	Section II.A.	Section II.B.	Section II.C.	Section III	Section III.A.	Section III.B.
1	Johnson and Johnson Official 7-Minute Workout	33.5	16.3	10.0	2.8	3.5	17.2	9.3	3.2	4.7	.	.	.
2	Nike+ Training Club	32.6	10.0	2.0	3.2	4.8	19.5	4.7	6.2	8.7	3.1	1.0	2.1
3	Running for Weight Loss: Interval Training	32.6	26.7	10.0	7.9	8.8	.	.	.	.	3.5	1.0	2.5
4	Fitness Buddy Free	31.0	8.1	2.0	3.6	2.5	22.9	8.7	8.6	5.7	.	.	.
5	FitnessBuilder	28.3	.	.	.	.	21.2	8.7	8.0	4.5	7.1	4.3	2.8
6	JEFIT	27.5	8.4	2.0	2.9	3.5	16.3	5.3	6.5	4.5	2.8	1.0	1.8
7	Body Space	26.2	9.0	2.7	3.5	2.8	14.9	6.0	5.5	3.3	2.4	1.0	1.4
8	Daily Workouts Free	25.5	13.0	5.3	4.1	3.5	12.5	6.0	3.7	2.8	.	.	.
9	StrongLifts 5x5	24.0	.	.	.	.	24.0	8.7	8.3	7.0	.	.	.
10	Jillian Michaels Slim Down	19.9	.	.	.	.	19.9	9.3	5.0	5.5	.	.	.
11	Fitness Point-Workout Exercise	19.0	.	.	.	.	19.0	10.0	5.7	3.3	.	.	.
12	C25K-5K Trainer Free	17.7	17.7	2.7	7.2	7.8	.	.	.	.	.	.	.
13	7 Minute Workout	17.5	8.7	2.7	3.7	2.3	8.8	2.7	3.4	2.7	.	.	.
14	Sworkit	17.5	7.0	2.0	2.7	2.3	8.0	2.7	3.1	2.2	2.5	1.0	1.5
15	7- Minute Workout- Fitness for Women	16.9	7.0	2.0	2.8	2.2	9.9	2.7	3.4	3.8	.	.	.
16	Abs Workout: Get Your Six Pack	14.9	.	.	.	.	14.9	9.3	3.2	2.3	.	.	.
17	Daily Butt Workout Free	14.9	.	.	.	.	14.9	8.0	3.9	3.0	.	.	.
18	Instant Abs Trainer	12.2	.	.	.	.	12.2	4.0	4.4	3.8	.	.	.
19	FitStar	11.3	.	.	.	.	8.9	2.7	2.8	3.5	2.4	1.3	1.1
20	Daily Ab Workout Free	11.3	.	.	.	.	11.3	6.7	2.7	2.0	.	.	.
21	Workout Trainer	10.0	.	.	.	.	10.0	2.7	4.2	3.2	.	.	.
22	Runtastic Six Pack Abs Trainer	9.1	.	.	.	.	9.1	2.0	5.0	2.2	.	.	.
23	The 7 Minute Workout- Get Fit	8.4	.	.	.	.	8.4	2.7	3.2	2.5	.	.	.
24	Cardio-Heart Rate Monitor + 7 Minute Workout	8.4	.	.	.	.	8.4	2.0	3.0	3.3	.	.	.
25	Belly Fat Workout Free	8.3	.	.	.	.	8.3	2.7	2.8	2.8	.	.	.
26	Strava Running and Cycling	8.0	8.0	2.7	2.5	2.8	.	.	.	.	.	.	.
27	Simply Yoga Free	6.4	.	.	.	.	.	.	.	.	6.4	3.3	3.1
28	Daily Yoga-Lose Weight, Get Relief	6.2	.	.	.	.	.	.	.	.	6.2	3.3	2.8
	**Mean**	**17.8**	**11.7**	**3.8**	**3.9**	**3.9**	**13.9**	**5.5**	**4.6**	**3.8**	**4.0**	**1.9**	**2.1**
	**Standard Deviation**	**8.9**	**5.9**	**3.0**	**1.8**	**2.2**	**5.2**	**2.9**	**1.8**	**1.6**	**1.9**	**1.3**	**0.7**

Note:
1. The highest possible overall score is 70; the highest possible sections I and II score is 30; the highest possible section III score is 10.
2. Section I is Aerobic Exercise; Section II is Strength and Resistance; Section III is Flexibility.
3. Sub-section A is Safety; Sub-section B is Program Principles; Sub-section C is Single Training Session Principles.
